# Investigation of cracks in GaN films grown by combined hydride and metal organic vapor-phase epitaxial method

**DOI:** 10.1186/1556-276X-6-69

**Published:** 2011-01-12

**Authors:** Jianming Liu, Xianlin Liu, Chengming Li, Hongyuan Wei, Yan Guo, Chunmei Jiao, Zhiwei Li, Xiaoqing Xu, Huaping Song, Shaoyan Yang, Qinsen Zhu, Zhanguo Wang, Anli Yang, Tieying Yang, Huanhua Wang

**Affiliations:** 1 Key Laboratory of Semiconductor Materials Science, Institute of Semiconductors, Chinese Academy of Sciences, P. O. Box 912, Beijing 100083, People's Republic of China; 2 Beijing Synchrotron Radiation Facility, Institute of High Energy Physics, Chinese Academy of Sciences, P. O. Box 918, Beijing 100039, People's Republic of China

## Abstract

Cracks appeared in GaN epitaxial layers which were grown by a novel method combining metal organic vapor-phase epitaxy (MOCVD) and hydride vapor-phase epitaxy (HVPE) in one chamber. The origin of cracks in a 22-μm thick GaN film was fully investigated by high-resolution X-ray diffraction (XRD), micro-Raman spectra, and scanning electron microscopy (SEM). Many cracks under the surface were first observed by SEM after etching for 10 min. By investigating the cross section of the sample with high-resolution micro-Raman spectra, the distribution of the stress along the depth was determined. From the interface of the film/substrate to the top surface of the film, several turnings were found. A large compressive stress existed at the interface. The stress went down as the detecting area was moved up from the interface to the overlayer, and it was maintained at a large value for a long depth area. Then it went down again, and it finally increased near the top surface. The cross-section of the film was observed after cleaving and etching for 2 min. It was found that the crystal quality of the healed part was nearly the same as the uncracked region. This indicated that cracking occurred in the growth, when the tensile stress accumulated and reached the critical value. Moreover, the cracks would heal because of high lateral growth rate.

## Introduction

Group III nitrides are attracting much attention for short-wavelength light emitters and high-temperature electronic devices. Nitride-based devices are mostly heteroepitaxially grown on non-native substrates, such as sapphire (Al_2_O_3_), Si, GaAs, and SiC. The differences of thermal expansion coefficient (TEC) and lattice constant between GaN and foreign substrates usually induce a large residual stress in thick GaN films. Homoepitaxy is very essential to improve the crystal quality. Hydride vapor-phase epitaxy (HVPE) is a promising technique for growing thick GaN film at reasonable cost. The conventional method of growing high quality thick film needs two systems. Before depositing the thick layer by HVPE, a template has been predeposited by MOCVD [1]. Compared with the conventional growth method, the combined hydride and metal organic vapor-phase epitaxial (MOCVD-HVPE) in one chamber has several great advantages: (1) the MOCVD and HVPE run in the same reactor without time-consuming modification or equipments replacement; (2) furthermore, the cracks and contamination introduced in the course of transfer can be voided; and (3) the growth methods can be alternated if necessary.

However, cracks are often produced in GaN thick film grown by HVPE. There are several intriguing aspects for the observed cracks of GaN on sapphire substrates. Itoh et al. [2] proposed that the cracks originated from the static cooling process. As the thermal expansion coefficient of GaN is smaller than that of sapphire [3], the film will suffer from biaxial compressive stress during cooling. Etzkorn and Clarke [4] also observed cracks in GaN film deposited by HVPE on SiC substrate. In our article, the cracks existing in GaN thick films were observed directly and the probable formation mechanism was proposed.

### Experiments

**Figure 1:**
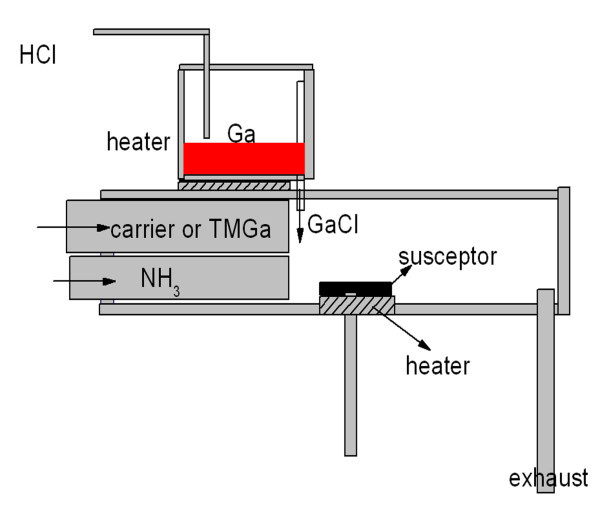
**MOCVD-HVPE main reactor**.

The sample was grown using a homemade MOCVD-HVPE system, as shown in Figure 1. The reactor system consists of two temperature zones which are heated by resistance wire heater. The liquid gallium (Ga) was heated to 900°C by the first heater for reacting with hydrogen chloride (HCl); the substrate was heated up to 1050°C using the second heater. Before depositing GaN thick film, a 60-nm thick low temperature (550°C) GaN buffer layer and a 0.82-μm thick high temperature layer were predeposited on a c-plane sapphire substrate by MOCVD. Ammonia (NH_3_) and trimethylgallium (TMGa) were used as N and Ga sources with the flow rate of 0.18 mol min^-1 ^and 50 μmol min^-1^, respectively. In addition, N_2 _was used as carrier gas with the flow rate of 2 standard liters per minute (SLM). In the HVPE experiments, GaCl was formed by the reaction of gaseous HCl and liquid Ga at 900°C, and then reacted with NH_3 _to grow GaN thick film. A 22-μm thick film was deposited by HVPE, with the HCl flow rate being 50 standard cubic centimeters per minute (sccm), NH_3 _flow rate being 4 SLM, and the N_2 _carrier gas flow rate being 2 SLM.

The high-resolution X-ray diffraction (D8 discover) was used to determine the lattice constant *c *near top surface. The curvature was also determined by this equipment, following the suggestion given by Liu et al. [5]. D8 discover was equipped with a twofold hybrid monochromatic and a threefold Ge (220) analyzer. The crystal quality of the sample was characterized using the high-resolution X-ray diffraction apparatus at Beijing Synchrotron Radiation Facility. The micro-Raman measurements were done using JYHR800 Raman spectrum. The laser was an argon ion laser operating at 514.5 nm. The spectral frequency resolution was less than 0.2 cm^-1 ^and the spatial resolution was less than 1 μm. The error bar is 0.2 cm^-1^. All micro-Raman spectra were recorded in the backscattering geometry. The spectrometer was calibrated using single-crystal silicon as a reference. The surface morphology and cracks were observed by SEM (using Hitachi S4800). The cathodoluminescence (CL) was performed in a scanning electron microscope (SEM) using Gantan mono CL system at room temperature.

## Results and discussion

**Figure 2:**
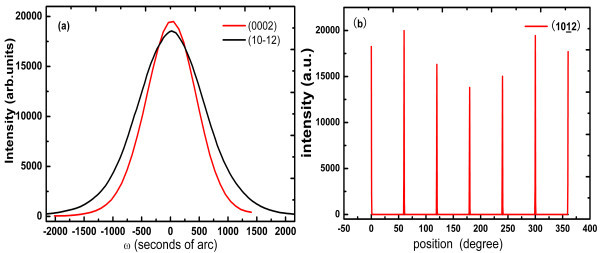
**The plots of XRD**. **(a) **XRD rocking curves of the (0002) and (1012) planes and **(b) **the PHI scan curve.

The overall crystal quality of the sample was determined by high-resolution X-ray diffraction with Synchrotron Radiation as light source. As illustrated in Figure 2a, the rocking curves of (0002) and (1012) were obtained and the full widths at half maximum (FWHM) were 970 and 1358 arc seconds, respectively. The phi scan presents a sixfold symmetry of wurtzite structure of GaN, as shown in Figure 2b. The dislocation density of the crystal was about 2 × 10^9 ^cm^-2 ^determined by XRC and AFM after selective etching [6,7].

**Figure 3:**
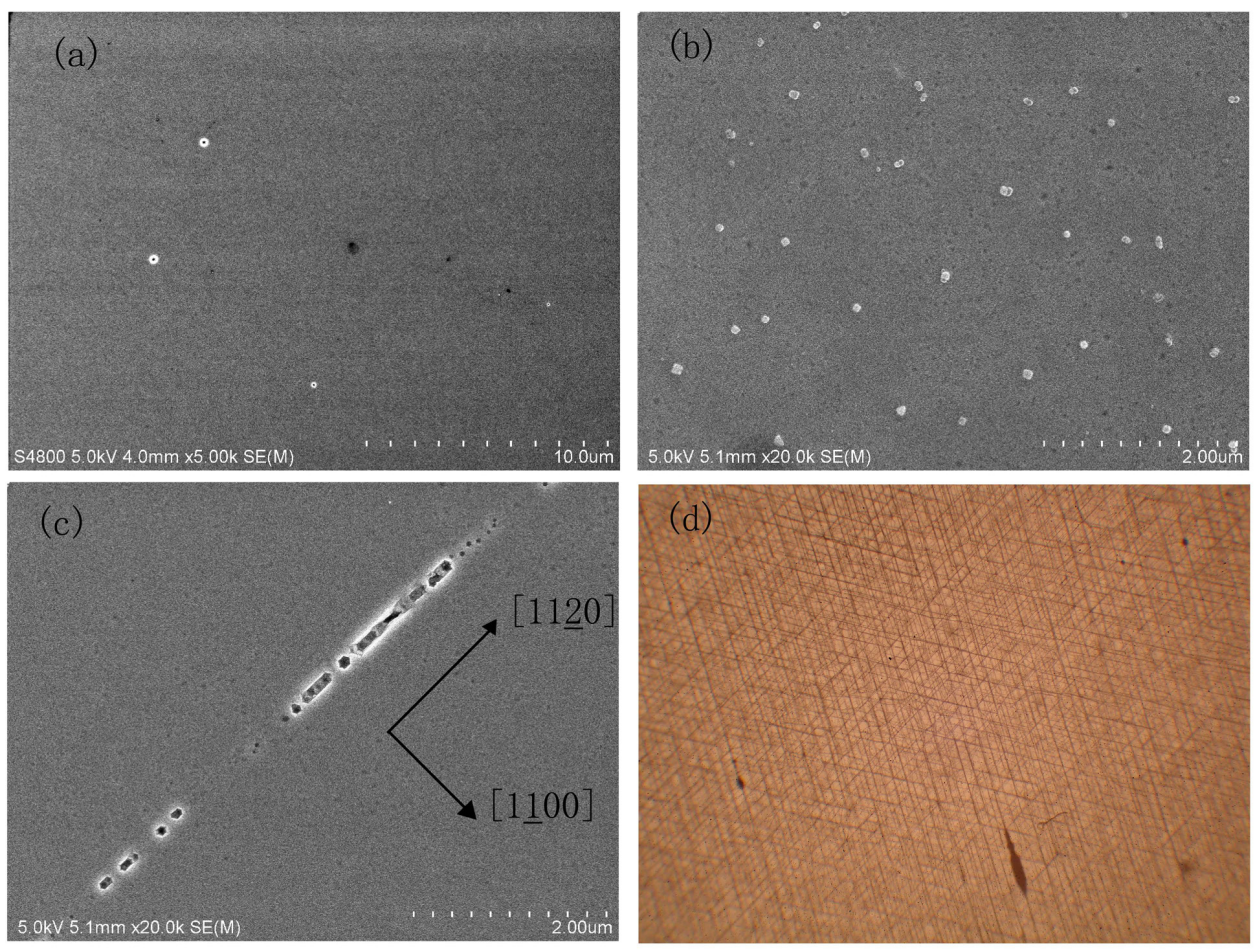
**SEM images of the GaN surface morphologies**. The etching time is **(a) **0 min **(b) **5 min, and **(c) **10 min. The cracks extend along the (1010) plane. **(d) **This is an optical micrograph of the cracks. This image is a transmitted image (10.5 mm × 8.5 mm).

**Figure 4:**
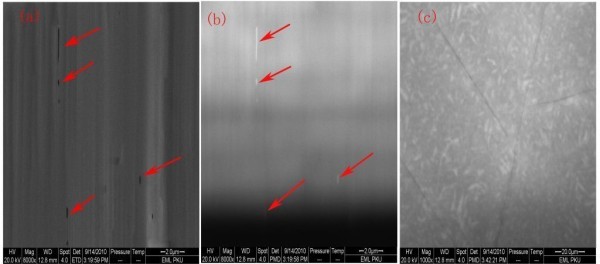
**The determination of the CL**. **(a) **The cross-sectional image of SEM. **(b) **Panchromatic CL cross-sectional image of epitaxial layer grown by MOCVD-HVPE, the white lines noted by red arrow line are cracks. (a) and (b) were taken simultaneously. **(c) **The panchromatic CL image of the sample etched for 10 min.

In order to observe the cracks under the surface, the sample was etched for 5 and 10 min in a solution of H_2_SO_4_:H_3_PO_3 _(3:1) at 200°C, and the two samples were marked as Af5 and Af10, respectively. The unetched sample was labeled as A. The etching rate was about 0.2 μm min^-1^. Cracks were observed on surface till the sample was etched for 10 min, as illustrated in Figure 3c. The underlayer cracks were also observed by optical microscope, as illustrated in Figure 3d. In the transmitted light image, the cracks were parallel to {1010} plane and formed a network arranged at 120° with each other. The effects of grain boundaries and dislocations have been revealed by CL mapping. The crystal quality of the grain boundaries is inferior to the other regions. High density of dislocations and other extended defects exist at the grain boundaries. If the cracks were located near the grain boundaries, various brightness distributions would exist between the cracked regions and the far away cracked regions [8,9]. As shown in Figure 4a, c, the bright distribution near the cracked regions and regions far away from cracks was nearly the same. We believe that the dislocations and grain boundaries do not interact with the cracks. This conclusion is also consistent with Figure 6b.

**Figure 5:**
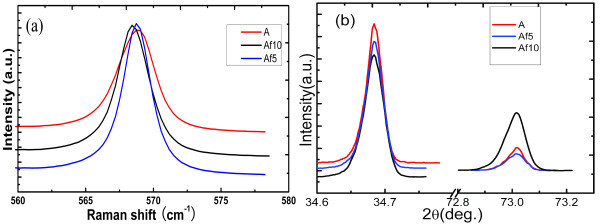
**The stress states of the top surface were determined by**: **(a) **the Raman frequency of the E2 (high), **(b) **the diffraction peaks of (0002) and (0004) determined by XRD in the *θ*-2*θ *mode.

The stresses were determined by HR-XRD and Raman, as shown in Figure 5. The lattice constant *c *is calculated by [10]

(1) $$d_{0001}=\frac{2\lambda }{2\sin(\theta_{0002}+\Delta \theta )}=\frac{4\lambda }{2\sin(\theta_{0004}+\Delta \theta )},$$

where *d*_0001 _equals to the lattice constant *c*; λ is the wavelength of the X-ray; *θ*_0002 _and *θ*_0004 _are the (0002) and (0004) plane diffraction peaks, respectively. The curves were determined using *θ*-2*θ *mode. Δ*θ *is zero error. The strain along the *c *direction is expressed as

(2) $$\varepsilon_{zz}=\frac{c-c_{0}}{c_{0}}$$

According to the relationship between the strain and stress [11], the stress in the plane can be expressed as

(3) $$\sigma_{xx}=-\frac{E}{2\nu }\varepsilon_{zz},$$

where *c*_0 _is the lattice constant of stress-free GaN. At room temperature, the free-stress lattice constant *c*_0 _is referenced to 0.51850 nm [12]. *σ*_*xx *_and ε_*zz *_are the biaxial stress and strain in the growth plane, respectively. *E *and ν are Young's modulus and Poisson's ratio, respectively. The determined and concluded data are shown in Table 1. These results suggest that the stress decreases with increasing etching time. The values of the stress and curvature in A and Af5 are nearly the same. However, the lattice constant *c *and the curvature rapidly changed in Af10.

**Table 1:** The lattice constant determined by XRD

	Degree		Lattice constant *c *(nm)	***ε***_**zz**_	***E***_**2 **_**(high) (cm^-1^)**	Radius of curvature (m)
					
	(0002)	(0004)				
A	34.6848	73.0178	0.51870	0.000386	568.735	0.98
Af5	34.6851	73.0176	0.51869	0.000366	568.711	0.98
A10f	34.6844	73.0174	0.51858	0.000154	568.504	1.02

The Raman shift at the near surface.

The Raman scattering is a useful tool for investigating the strain of epitaxial film. The frequency of *E*_2 _(high) phonon is very sensitive to the in-plane strain. As illustrated in Figure 5a, the frequencies of *E*_2 _high exhibit redshift with increasing etching time, which is consistent with the variations of lattice constant c. Many articles reported that the strain-free frequency of *E*_2 _high mode was 567.5, 567.6, and 568 cm^-1 ^[13-15]. If the frequency is larger than the value of reference, the presence of compressive stress will be expected; on the other hand, the stress will be tensile. This effect had already been observed for hydrostatic pressure, biaxial strain, and uniaxial strain [11]. The obtained lattice constants *c *were larger than the reference value, so the samples suffered from compressive stress at room temperature. The variation of the stress which was calculated along depth was in agreement with the shift of *E*_2 _(high) phonon frequency. Furthermore, this trend was consistent with the variation of the curvature. Sample Af10 had two notable features: the cracks were observed in the surface; stress rapidly dropped. We could believe that the stresses were mainly relaxed by producing cracks.

**Figure 6:**
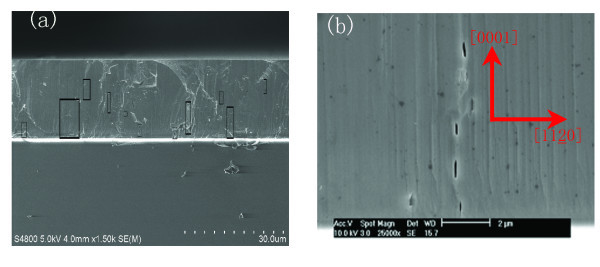
**Cross-sectional SEM images and the cleavage plane is (1010)**. **(a) **The cross-section was unetched, the black frame indicates the cracks. **(b) **The cross-section was etched for 2 min.

In order to gain further insight into the nature of the cracks, we observed the cross-sections of films after cleaving. The cleavage plane was (1010). A typical cross-sectional SEM image was shown in Figure 6a. The cracks marked in black frame had a number of notable features: the cracks were perpendicular to the film/substrate interface; the cracks neither approached to the surface nor extended to the substrate; and the cracks appeared to be pinched off at several locations, and with well-rounded ends, suggesting the cracks may heal up.

**Figure 7:**
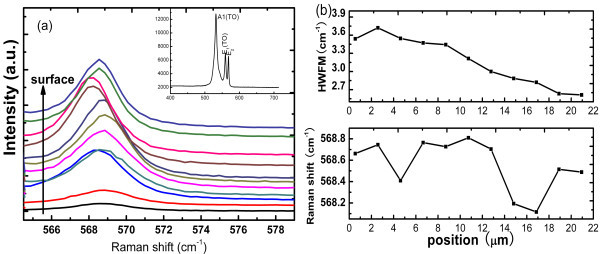
**The determination of Raman**. **(a) **Micro-Raman spectra of the cross-section are obtained by scanning from the bottom of the interface to the surface. **(b)**The phonon frequencies of the E2 (high) and FWHM vary with depth.

In order to gain a deep insight into the origin of the cracks, stress distribution along depth was measured by cross-sectional micro-Raman spectral. Raman spectra were conducted in 2 μm steps along the depth. The frequencies and the linewidths of the Raman mode were shown as a function of the distance from the interface of GaN/substrate as shown in Figure 7b. It was noticeable that the peaks of Raman *E*_2 _(high) phonon mode were variable; it blueshifts at the interface of film/substrate, then goes to steadiness in the following, after that the peaks fall down and then returned to blueshift; the linewidth of the *E*_2 _phonon was approximately 2.6 cm^-1 ^near the surface and increased with decreasing distance from the interface of film/substrate. The linewidths were mainly affected by stress and defects. Many articles reported that columnar structures and defects existed at the near interface region [8,16]. It is reasonable to conclude that the broader *E*_2 _linewidth near the interface is due to the disorder and strain associated with these defects. With increasing thickness, the crystal quality gets better. This result is in agreement with the variation of the linewidths.

The determined stress is the sum of the intrinsic and extrinsic at room temperature. The stress is affected by lattice mismatch, coefficient of thermal expansion mismatch, islands coalescence, grain growth, and gas impurity [17]. Thermal strains induced by the expansion coefficient difference between the substrate and GaN film dominate in the extrinsic stress. This strain is expressed as

(4) $$\varepsilon_{\text{t}}=\int_{T_{\text{room}}}^{T_{\text{growth}}}\left(\alpha_{\text{f}}\left(T\right)-\alpha_{\text{s}}\left(T\right)\right)\;\text{d}T,$$

where α_f_(*T*) and α_s_(*T*) are the thermal expansion coefficients of the film and substrate, respectively. α_s_(*T*) is larger than α_f_(*T*) [18]. The film/substrate system reduces their elastic potential energy by bending, resulting in a strain gradient along the depth. It is assumed that the stress distribution in the substrate and film is a linear function along the depth. The elastic energy in the *z*th layer *U*(*z*) caused by bending is given by [19]

(5) U(z)={Mf(ε0−kz+εt)2,hs2<z<hf+hs2Ms(ε0−kz)2,−hs2<z<hs2,

where *h*_f _and *h*_s _are the thicknesses of the film and the substrate and their values are 22 and 430 μm, respectively. *z *is the distance from the bottom of the substrate. *M*_s _and *M*_f _are the elastic constants of the substrate and film, respectively. *ε*_0 _is the strain in the central plane of substrate. The system potential energy *V*_system _is

(6) Vsystem=∫-hs2hs2+hfU(z) dz.

**Figure 8:**
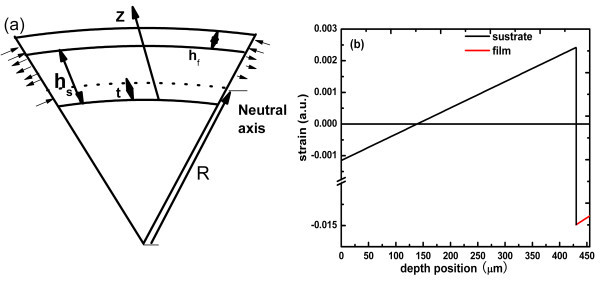
**The schematic diagram of**: **(a) **the bending in the GaN/Al_2_O_3_, induced by the difference in the thermal expansion coefficients. **(b) **The strain distribution with the depth by calculation.

Based on energy minimization principle, *k *and *ε*_0 _can be obtained by ∂*V*/∂ε_0 _= 0 and ∂*V*/∂*k *= 0, We defined *r *as the weighted ratio of elastic constant of the film and the substrate.

$$r=\frac{M_{\text{f}}h_{\text{f}}}{M_{\text{s}}h_{\text{s}}}$$

and

(7) $$k=\frac{6e_{\text{t}}r}{h_{\text{s}}}\left(1+\frac{h_{\text{f}}}{h_{\text{s}}}\right){\left[1+4r+6r\frac{h_{\text{f}}}{h_{\text{s}}}+4r{\left(\frac{h_{\text{f}}}{h_{\text{s}}}\right)}^{2}+r^{2}{\left(\frac{h_{\text{f}}}{h_{\text{s}}}\right)}^{2}\right]}^{-1},$$

(8) $$\varepsilon_{0}=-\varepsilon_{\text{t}}r\left(1+r{\left(\frac{h_{\text{f}}}{h_{\text{s}}}\right)}^{2}\right){\left[1+4r+6r\frac{h_{\text{f}}}{h_{\text{s}}}+4r{\left(\frac{h_{\text{f}}}{h_{\text{s}}}\right)}^{2}+r^{2}{\left(\frac{h_{\text{f}}}{h_{\text{s}}}\right)}^{2}\right]}^{-1},$$

where *M*_f _and *M*_s _are the elastic constants and can be calculated by

(9) $$M=C_{xx}+C_{xy}-\frac{2C_{xz}^{2}}{C_{zz}},$$

where *C*_*ij *_are the stiffness constants (as shown in Table 2) and the *x*, *y*, and *z *axes are chosen along the GaN 1120, 1100, and 0001 directions, respectively. Since these directions are parallel to the crystal principal axis, the shear stress components (*i *≠ *j*) are zero. The values of *M*_f _and *M*_s _are 455 and 603 GPa, respectively. We calculated that the radius of curvature was 0.123 m and *ε*_0 _was 6.4 × 10^-4^, assuming the film and the substrate were not in the plastically deforming area in the cooling process. As illustrated in Figure 8b, the largest tensile strain is 0.0025 located at substrate side near the interface and whole GaN film suffers from the compressive stress. If cracking happened in the cooling process, it would be difficult to explain why the cracks did not appear in the substrate but in the film. It is reasonable to believe that the cracks are generated in the growth process.

**Table 2:** The value of elastic stiffness tensor elements *C*_*i*__*j*_

	*C*_*xx *_(Gpa)	*C*_*xy *_(Gpa)	*C*_*xz *_(Gpa)	*C*_*zz *_(Gpa)	References
GaN	374	106	70	379	[26]
Al_2_O_3_	409.2	165.4	113.0	490.2	[27]

The cracks nucleation and extension are the consequences of both the existence of tensile stress and exceeding the critical thickness during the growth process. We should explain the origin of the tensile stress. Many authors observed intrinsic tensile stress using in situ measurements of wafer bending curvature [20-22]. They found that the compressive stress appeared first, and then the compressive stress switched to steady tensile stress. This tensile stress was attributed to islands coalescence. This phenomenon was independent of the substrate. Hoffman [23] proposed that adjacent islands with vertical surface grew closer to one another and then elastically snapped together when the gap between the islands reached some critical size. The decrease of the solid-vapor interfacial energy balances the increase of the stress-related mechanical energy and grain boundary-related surface energy. Cracks will occur once the stressed films reach the critical thickness. Once the cracks have been introduced, an opening channel would be left. However, it is difficult to explain that the cracks do not extend to the surface and many cracks are buried in the consequence growth. Etzkom and Clarke [4] proposed several possibilities for the cracks that get closed up and buried: film lateral growth at the crack opening; concurrent diffusion transport by surface diffusion, driven by capillarity; and cracks face touch in cooling process. However, only at high temperature the atom have high diffusing rate. In our system, the temperature dropping from 1050 to 600°C only needs 3 min. Some authors had calculated the Ga atom surface diffuse length and the value was less than 13 nm min^-1 ^at 1050°C [24]. A large number of Ga and N atoms concurrent diffusion along the cracks surface are very difficult. If the healed part originates from the face touch in cooling, the crystal quality would be inferior to the uncracked part. In order to compare crystal quality of the cracks edge with that of healed part, the cross was etched for 2 min at 200°C in mixed solution of H_2_SO_4_:H_3_PO_3 _(3:1). Only crack edges were etched faster than those of the healed part, as shown in the Figure 6b. It would be concluded that the lateral growth predominates in the healing process. The tensile stress was mainly relaxed by the cracks, but residual tensile stress also was present in the uncracked region [25]. The cracking will be reproduced in the uncracked region.

When the temperature dropped from the growth temperature to the room temperature, the thermal stress mostly exerted in the healed apartment and uncracked region. These explanations are consistent with the result of Raman spectra in Figure 7b and surface stress analysis in Table 1. The variation of phonon frequency appeared as S-shaped distribution along depth; the cracks did not extend to the surface or approach the substrate; the crystal quality of healed part is comparable with the uncracked part.

## Conclusion

The origin of cracks in GaN film grown by MOCVD-HVPE system has been analyzed by SEM, HR-XRD, Raman, and CL. The stress distribution was obtained by cross-sectional Raman spectra. According to the stress distribution and the cracks distribution, it would be expected that the cracks originate from the growth process. When the films reach the critical thickness, cracks will be generated. Then the cracks will be healed in the consequent growth by lateral growth. So the cracks do not extend to either the substrate or the film surface.

## Abbreviations

CL: cathodoluminescence; Ga: gallium; HCl: hydrogen chloride; HVPE: hydride vapor-phase epitaxy; MOCVD: metal organic vapor-phase epitaxy; NH_3_: ammonia; SEM: scanning electron microscopy; SLM: standard liters per minute; sccm: standard cubic centimeters per minute; TEC: thermal expansion coefficient; TMGa: trimethylgallium; XRD: X-ray diffraction.

## Competing interests

The authors declare that they have no competing interests.

## Authors' contributions

JL carried out the experiments and measured the material, drafted the manuscript. XL, SY, QZ and ZW directed the experiments and the drafting of the paper. CL and YG participated the growth of material. ZL and XX carried out the measurement of Raman. TY and HW carried out the measurement of XRD. AY and HS carried out the etching. HW and CJ carried out the measurement of CL.
